# Autoantibody Signature Differentiates Wilms Tumor Patients from Neuroblastoma Patients

**DOI:** 10.1371/journal.pone.0028951

**Published:** 2011-12-16

**Authors:** Jana Schmitt, Andreas Keller, Nasenien Nourkami-Tutdibi, Sabrina Heisel, Nunja Habel, Petra Leidinger, Nicole Ludwig, Manfred Gessler, Norbert Graf, Frank Berthold, Hans-Peter Lenhof, Eckart Meese

**Affiliations:** 1 Department of Human Genetics, Medical School, Saarland University, Homburg, Germany; 2 Department of Paediatric Oncology and Haematology, Medical School, Saarland University, Homburg, Germany; 3 Developmental Biochemistry, Biocenter, University of Wuerzburg, Wuerzburg, Germany; 4 Department of Paediatric Oncology and Haematology, University of the Cologne, Cologne, Germany; 5 Center for Bioinformatics, Saarland University, Saarbruecken, Germany; Hospital Infantil Universitario Niño Jesús, Spain

## Abstract

Several studies report autoantibody signatures in cancer. The majority of these studies analyzed adult tumors and compared the seroreactivity pattern of tumor patients with the pattern in healthy controls. Here, we compared the autoimmune response in patients with neuroblastoma and patients with Wilms tumor representing two different childhood tumors. We were able to differentiate untreated neuroblastoma patients from untreated Wilms tumor patients with an accuracy of 86.8%, a sensitivity of 87.0% and a specificity of 86.7%. The separation of treated neuroblastoma patients from treated Wilms tumor patients' yielded comparable results with an accuracy of 83.8%. We furthermore identified the antigens that contribute most to the differentiation between both tumor types. The analysis of these antigens revealed that neuroblastoma was considerably more immunogenic than Wilms tumor. The reported antigens have not been found to be relevant for comparative analyses between other tumors and controls. In summary, neuroblastoma appears as a highly immunogenic tumor as demonstrated by the extended number of antigens that separate this tumor from Wilms tumor.

## Introduction

Neuroblastoma is the most common childhood cancer occurring in about 7% of childhood cancers, and has an incidence of about 10 per million children per year in Europe [1]. Neuroblastoma is a clinically very heterogenous tumor that was originally classified into six different stages according to the INRG (I, IIA, IIB, III, IV, IVS) by the postsurgical INSS [2]. A new pretreatment staging system, the INRG staging system (INRGSS), was developed in 2009 [3]. By now, stage, age, histologic category, grade of tumor differentiation, MYCN status, 11q aberration and ploidy are the most important parameters for pretreatment risk classification [4]. The most prominent genetic marker is the MYCN-amplification that has been associated with a worse prognosis [5], [6]. MYCN that is located on 2p23-24 encodes proteins deregulating cell growth and proliferation upon amplification. Further amplifications in neuroblastoma include the MDM2 gene on 12q13 and the MYCL gene at 1p32 [7], [8]. Furthermore, deletions and loss of heterozygosity (LOH) of chromosome 1p seem to be significant for prognosis [9]–[11].

Urinary homovanillic acid and vanillylmandelic acid as metabolites of catecholamines [12] have been employed in mass screenings for neuroblastoma in Japan, North America and Europe [13]–[16]. These screenings increased the incidence in infants without decreasing the incidence of unfavorable advanced-stage disease in older children. Overall, mass screenings did not reduce the mortality for neuroblastoma [16], [17]. As of now, the effectiveness of mass screening is controversially discussed [18]–[21].

A specific diagnostic challenge is the differentiation between neuroblastoma and Wilms tumor (WT) as the most common renal childhood tumor [22]. There is evidence that preoperative imaging for differentiation between Wilms tumors and Non-Wilms tumors is not in 100% accurate [23], [24]. In Europe Wilms tumor patients are treated without histology on the basis of their characteristic radiological features alone according to the Societé Internationale d'Oncologie Pédiatrique (SIOP) protocols. Characteristic autoantibody signatures may be useful to confirm the radiological discrimination between the suspected Wilms tumor and e.g. neuroblastoma. In the US all WT patients undergo histological confirmation and autoantibody signatures are therefore not needed for differential diagnosis (Children's Oncology Group) [25], [26].

As of now, most classifications with autoantibodies were designed to separating adult cancer patients from healthy controls [27]–[30]. The accuracy of such separations yielded average values of 80–95%. There are only few attempts to define pattern of immunogenic antigens that allow classifications between different diseases. A classification of glioma sera versus sera of patients with other intracranial tumor yielded an accuracy of 88.0%. A classification between glioma sera and sera of patients with non-tumor brain pathologies yielded an accuracy of 87.8% [31]. Lung cancer and patients with non-tumor lung pathologies were separated with an accuracy of 88.5% [28]. As of now, there have not been any reports on autoantigen signatures in renal childhood tumors or neuroblastoma.

In this work, we investigated to what extend the humoral immune response can be exploited to arrive at new biological markers that may be useful for children with an abdominal mass. Specifically, we ask if and how many autoantibodies can be found in children with neuroblastoma, if an autoantibody signature can be deduced for neuroblastoma and if such a signature allows differentiation between neuroblastoma and Wilms tumor. This study is aimed to lay the ground to further exploit autoantibody signatures for diagnostic purposes of patients with neuroblastoma.

## Results

### Search for immunogenic antigens of patients with Wilms tumors or neuroblastomas

To identify immunogenic clones that differentiate Wilms tumor patients from patients with neuroblastoma we used an array based approach as previously described [29], [32]. The array encompassed 1,827 immunogenic clones including 509 human in-frame peptides and 1318 out-of-frame sequences. While in-frame clones are transcribed in the correct reading frame, out of frame clones may have an amino-acid sequence other than the natural antigen. Each of the clones expressed a recombinant protein that was previously shown to react with autoantibodies of human sera.

In this multicenter study we screened the arrays with sera of neuroblastoma patients and Wilms tumor patients. The reactivity of serum autoantibodies against the *E. coli* expressed clones was measured by an automated image analysis system. We discounted clones that yielded read-out intensity values below 50. After eliminating these clones we obtained 1520 reactive clones including 422 in-frame clones. To differentiate between neuroblastoma and Wilms tumor patients, we separately analyzed patients prior to treatment and after treatment. First, we compared 30 sera of untreated neuroblastoma patients and 53 sera of untreated Wilms tumor patients. For both diseases, the majority of the clones reacted with approximately 50% of the sera. Only a smaller number of clones reacted with nearly all sera tested. Likewise, a smaller number reacted with few sera. Table 1 shows the distribution of the frequency of reactivities for all clones. Overall, we found for both Wilms tumor patients and neuroblastoma patients an extended and comparably high number of serum antibodies that react with the recombinant antigens.

**Table 1:** Number of reactive clones with sera of untreated neuroblastoma patients (NBs) and sera of untreated Wilms tumor patients (WTs).

	Sera of untreated WTs		Sera of untreated NBs		Sera of treated WTs		Sera of treated NBs	
%	all clones	in frame	all clones	in frame	all clones	in frame	all clones	in frame
0.00–10.00	27	9	47	12	11	6	28	8
10.01–20.00	69	29	111	29	88	25	114	34
20.01–30.00	143	53	137	36	131	53	143	37
30.01–40.00	182	71	202	51	215	74	168	49
40.01–50.00	203	53	213	68	186	62	197	53
50.01–60.00	222	56	200	63	277	76	226	64
60.01–70.00	225	69	213	54	194	41	228	70
70.01–80.00	221	44	193	59	223	52	197	55
80.01–90.00	156	28	132	35	121	24	151	39
90.01–100.00	71	**8**	71	**14**	73	**8**	67	**12**

### Identification of specific clones that contributed most to a separation between patients with neuroblastoma or Wilms tumors

Beside the numerical similarity we frequently found the same antigens that were reactive against sera of neuroblastoma patients and sera of Wilms tumor patients. As an example we chose clones that were reactive with over 90% of the sera. We found 14 in frame clones that were reactive with more than 90% of sera from untreated neuroblastoma patients and eight in frame clones that were reactive with more than 90% of sera from untreated Wilms tumor patients. Five of these clones were reactive in over 90% of sera of both patients groups. Clones that were reactive with over 90% of either Wilms tumor or neuroblastoma patients, were each also reactive with 68.5–90% of sera of the other patient group (Tables 1 and 2).

**Table 2:** Frequency of clones that were highly reactive in Wilms tumor (WTs) /neuroblastoma patients (NBs).

	frequency			frequency	
	sera of	sera of		sera of	sera of
clone	untreated WTs	untreated NBs	clone	treated WTs	treated NBs
MPMGp800I14577	**0,944**	0.9	MPMGp800M16569	**0,943**	0,700
MPMGp800E17587	**0,925**	0.7	MPMGp800G07549	**0,943**	0,900
MPMGp800F10584	**0,907**	0.9	MPMGp800P23525	**0,914**	0,667
MPMGp800M18529	0,815	**1,000**	MPMGp800M03509	**0,914**	0,786
MPMGp800J02545	0,815	**1,000**	MPMGp800M18529	**0,914**	0,733
MPMGp800C11538	0,796	**0,967**	MPMGp800G09554	**0,914**	0,900
MPMGp800I19519	0,870	**0,967**	MPMGp800J02545	0,857	**0,967**
MPMGp800F09528	0,852	**0,933**	MPMGp800B14594	0,743	**0,967**
MPMGp800G09554	0,815	**0,933**	MPMGp800G24535	0,743	**0,967**
MPMGp800I07558	0,796	**0,933**	MPMGp800M08584	0,857	**0,933**
MPMGp800M16569	0,685	**0,933**	MPMGp800B12523	0,714	**0,933**
MPMGp800B12523	0,833	**0,933**	MPMGp800L20578	0,857	**0,933**
			MPMGp800G03526	0,600	**0,933**
			MPMGp800H19569	0,800	**0,933**
			MPMGp800K08584	0,600	**0,933**
			*MPMGp800I08563*	*0,429*	***0,933***

Clones reactive with either more than 90% of sera from untreated Wilms tumor patients or sera from untreated neuroblastoma patients and either of sera from treated Wilms tumor patients or sera from treated neuroblastoma patients (bold) and the appropriate frequency. Only one clone (italics) strongly differs in reaction frequency between both groups analyzed. Clones are ordered by frequency.

Based on the frequencies of seroreactivities we set out to identify specific clones that contributed most to a separation between neuroblastoma and Wilms tumors. To this extent we computed the AUC (area under the curve) value for each clone. Most of clones showed AUC values from 0.3 to 0.7 (Table 3). Clones with an AUC value greater than 0.7 or smaller than 0.3 were considered informative for the separation. Clones with AUC values smaller than 0.3 were more reactive with neuroblastoma sera and clones with AUC values greater than 0.7 were more reactive with Wilms tumor sera. For our analysis we concentrated on in-frame clones that encode known proteins. For out-of-frame clones, we cannot readily deduce the according proteins. Notably, we found a characteristic signature for neuroblastoma versus Wilms tumor only focusing on in-frame clones. In total, we obtained two in frame clones with AUC values greater than 0.7 and 16 in frame clones with AUC values smaller than 0.3 including two clones with values even smaller than 0.2. Both of the latter clones encoded the protein exostosin-2 (Table 4). The first exostosin-2 encoding clone showed an AUC value of 0.189 (p-value of 0.001) and the second clone an AUC value of 0.200 (p-value 0.0017). The seroreactivity of the first clone was 2.25 fold higher with neuroblastoma sera as compared to Wilms tumor sera and the second clone was 3.24 fold higher with neuroblastoma sera. A third clone encoding exostosin-2 was also informative with an AUC value of 0.269. This clone was 1.9 fold more reactive in neuroblastoma sera. Table 4 provides an overview of all reactive clones informative for the separation between neuroblastoma and Wilms tumor sera. We did not explore all 16 clones, because we concentrated on the clones with the most significant AUC value. These clones differed the most in reaction frequency between sera from neuroblastoma patients and sera from Wilms tumor patients. In general, we found that informative clones are more frequently reactive with sera of untreated neuroblastoma patients than with sera of untreated Wilms tumor patients indicating that the seroreactivity found in neuroblastoma patients contribute most to the difference in the antigen profile of these diseases.

**Table 3:** Distribution of AUC values for the classification of untreated Wilms tumor patients (WTs) vs. untreated neuroblastoma patients (NBs) and treated Wilms tumor patients (WTs) vs. treated neuroblastoma patients (NBs).

	untreated NBs vs.		treated NBs vs. treated	
AUC value	untreated WTs		WTs	
	all clones	in frame	all clones	in frame
0.9–1.0	0	0	0	0
0.8–0.9	4	0	0	0
0.7–0.8	55	2	16	1
0.6–0.7	290	44	201	36
0.5–0.6	495	121	494	108
0.4–0.5	439	135	488	147
0.3–0.4	208	102	256	98
0.2–0.3	26	16	55	29
0.1–0.2	2	2	9	3
0.0–0.1	0	0	0	0

**Table 4:** Antigens with a significant AUC value for the classification of untreated Wilms tumor patients vs. untreated neuroblastoma patients.

Antigen	AUC
**>0.7**	
*Amyloid beta A4 precursor protein-binding family B member 1*	*0,735*
Nonhistone chromosomal protein HMG-14	0,709
**<0.3**	
***Exostosin-2***	***0,189***
***Exostosin-2***	***0,200***
**Glial fibrillary acidic protein**	**0,227**
GRIP1 associated protein 1	0,244
ELAV-like protein 3	0,248
Secretogranin III	0,268
*Exostosin-2*	*0,269*
splicing factor proline/glutamine rich	0,271
Vimentin	0,272
Splicing factor proline/glutamine rich	0,278
Eukaryotic translation initiation factor 1	0,280
Peptidyl-prolyl cis-trans isomerase E	0,291
B-cell CLL/lymphoma 11A isoform 3	0,296
*Poly(A) binding protein cytoplasmic 1*	*0,296*
Protein NDRG1	0,298
RNA binding motif protein 6	0,298

The three most informative antigens (bold) and the antigens informative prior to and after chemotherapy (italics) are shown.

### Influence of treatment on the spectrum of clones that contributed most to a separation between neuroblastoma and Wilms tumors

Next we asked whether the treatment influenced the difference between the antigen profiles of neuroblastoma and Wilms tumors. To this end, we analyzed 30 sera of treated neuroblastoma patients, and 35 sera of treated Wilms tumor patients. Like for the untreated patients, we found that both treated Wilms tumor patients and treated neuroblastoma patients show a comparably high number of reactive serum antibodies. Likewise, two clones that were frequently reactive with untreated Wilms tumor sera were also frequently reactive with untreated neuroblastoma sera and vice versa. Most of clones that were reactive with over 90% of either treated Wilms tumor or treated neuroblastoma patients, were each also reactive with high percentage of sera of the other patient group (Table 2). Only one clone that was frequently reactive in sera from treated neuroblastoma patients (0.933) was much less reactive in sera of treated Wilms tumor patients (0.429) (Table 2 highlighted in yellow). This clone encodes the amyloid beta A4 precursor protein-binding family B member 1. AUC analysis revealed one in frame clone with a value greater than 0.7 and 29 in frame clones with values smaller than 0.3 including three clones with values smaller than 0.2. These three clones encoded ELAV-like protein 4, Microtubule-associated proteins 1A/1B light chain 3A precursor and amyloid beta A4 precursor protein-binding family B member 1, respectively (Table 5). As for the untreated patients, the seroreactivity found in treated neuroblastoma patients contributed most to the difference between the antigen profile of neuroblastoma and Wilms tumor patients. Notably among the clones informative for the treated patients was again a clone encoding exostosin-2 (AUC value 0.290). The seroreactivity of this clone was 2.5 fold higher with sera of treated neuroblastoma patients as compared to sera of treated Wilms tumor patients. Thus, an increased seroreactivity for exostosin-2 was found in neuroblastoma patients both prior and after treatment.

**Table 5:** Antigens with a significant AUC value for the classification of treated Wilms tumor patients vs. treated neuroblastoma patients.

Antigen	AUC
**>0.7**	
hairy and enhancer of split 5	0,702
**<0.3**	
**ELAV-like protein 4**	**0,159**
**Microtubule-associated proteins 1A/1B light chain 3A precursor**	**0,165**
**Amyloid beta A4 precursor protein-binding**	**0,175**
FK506-binding protein 3	0,204
Protein flightless-1 homolog	0,213
Pyruvate kinase isozymes M1/M2	0,216
*Poly(A) binding protein cytoplasmic 1*	*0,235*
STIP1 homology and U-box containing protein 1	0,237
40S ribosomal protein S8	0,240
40S ribosomal protein S6	0,245
8D6 antigen	0,246
*Amyloid beta A4 precursor protein-binding family B member 1*	*0,257*
60S ribosomal protein L8	0,267
Microtubule-associated protein 2 isoform 1	0,268
Metastasis associated protein	0,269
Minichromosome maintenance complex component 3	0,270
Ribosomal protein L37	0,270
Calsyntenin-1 precursor	0,272
*Amyloid beta A4 precursor protein-binding family B member 1*	*0,273*
Podocalyxin-like 2	0,276
Nonhomologous end-joining factor 1	0,276
Ribosomal protein S4 X-linked X isoform	0,277
Nuclear prelamin A recognition factor isoform c	0,279
Ribosomal L1 domain containing 1	0,283
STIP1 homology and U-box containing protein 1	0,283
Bromodomain-containing protein 7	0,285
40S ribosomal protein S6	0,287
*Exostosin-2*	*0,290*
Alveolar soft part sarcoma chromosome region	0,295
Sorbin and SH3 domain containing 2 isoform 2	0,297
Nuclease sensitive element binding protein 1	0,299

The three most informative antigens (bold) and the antigens informative both prior to and after chemotherapy (italics) are shown.

### Accuracy of the separation between untreated Wilms tumor patients versus untreated neuroblastoma patients and between treated Wilms tumor patients versus treated neuroblastoma patients

Besides the search for specific reactive antigens, we asked if and to what extend an autoantibody signature based on the identified in-frame antigens allows separation of both, untreated Wilms tumor patients versus untreated neuroblastoma patients and treated Wilms tumor patients versus treated neuroblastoma patients. For the separation of untreated Wilms tumor patients versus untreated neuroblastoma patients, we obtained an accuracy of 0.868 (95% Confidence Intervals (CI): [0.853–0.883]), a sensitivity of 0.870 (95% CI: [0.853–0.887]) and a specificity of 0.867 (95% CI: [0.843–0.891]. For this classification, we obtained a positive predictive value of 0.867, a negative predictive value of 0.870, a positive likelihood of 6.525 and a negative likelihood of 0.150. Comparable results were obtained for the classification between treated neuroblastoma and treated Wilms tumor patients. For this separation we found an accuracy of 0.838 (95% CI: [0.827–0.849]), a sensitivity of 0.817 (95% CI: [0.802–0.831]) and a specificity of 0.860 (95% CI: [0.843–0.877]) with a positive predictive value of 0.854, a negative predictive value of 0.824, a positive likelihood of 5.833 and a negative likelihood of 0.213. In summary, we found specific autoantibody signatures both for Wilms tumor and neuroblastoma. The classification accuracy was similar both prior and after treatment. The specific autoantigens that contributed to the classification were at least in part different prior and after chemotherapy (Table 4 und 5). But we showed that the treatment changes the autoantibody profiles, respectively.

## Discussion

Neuroblastoma and Wilms tumors are the most important abdominal tumors in small children. As they are situated nearby in the abdomen imaging studies are not able to distinguish them in all cases. Catecholamines are not always elevated in neuroblastoma and MIBG scanning is done with a radioactive substance. Diagnostic tools using radioactivity should always be avoided in small children, if possible. A good marker to discriminate all cases of Wilms tumor from neuroblastoma is not available, yet. It has also been shown by Hero et al. [24], that in few instances wrong treatment is given to some children with neuroblastoma, as their tumors were considered as nephroblastoma based on imaging studies. Therefore better biomarkers are needed. Our analysis shows that both neuroblastoma and Wilms tumor patients have numerous serum autoantibodies.

Highly informative for the classification of neuroblastoma versus Wilms tumor was the ELAV-like protein 4 (ELAVL4) that is implicated in neuronal differentiation and has been associated with Parkinson's disease [33], the Microtubule-associated proteins (MAP) 1A/1B light chain 3A precursor that are microtubule-associated and mediate the interactions between cytoskeleton and microtubules [34], Amyloid beta A4 precursor protein-binding family B member 1 (APBB1) that plays a role in controlling neurogenesis of GnRH-1 neurons [35].

Furthermore, among the informative antigens was exostosin-2 (EXT2) antigen that was most informative for the separation of untreated neuroblastoma patients from untreated Wilms tumor patients as determined by the AUC value. Likewise EXT2 was informative for the separation between treated neuroblastoma and treated Wilms tumor patients. In both comparisons the EXT2 seroreactivity was increased in frequency in neuroblastoma patients as compared to Wilms tumor patients. An autoantibody response against EXT2 has not been reported previously for other adult tumors according to Immunome Database (http://ludwig-sun5.unil.ch/CancerImmunomeDB/, last accessed in April 2011). The EXT2 gene encodes a glycosyltransferase that is involved in the heparan sulfate biosynthesis [36]–[38]. Loss of heterozygosity was described for multiple osteochondromas, also known as hereditary multiple exostoses [39]. A reduced level of EXT2 has been found in exostosis chondrocytes [36]–[40]. Based on these findings, EXT2 is discussed as a putative tumor suppressor. Although these data contribute to understanding the biological role of EXT2, they do not help to understanding the autoantibody response that we frequently found in neuroblastoma. Antigens may become immunogenic by fairly different mechanisms including overexpression, mutation or aberrantly degradation. Likewise post-translational modifications including glycosylation, phosphorylation, oxidation or proteolytic cleavage may play a role for proteins in becoming immunogenic by enhancing the self-epitope or generating a neo-epitope [41].

For diagnostic purposes, our analysis focuses on the comparison between neuroblastoma and Wilms tumors it is important to ask whether the antigens that contribute best to this separation are also frequently immunogenic in other tumor diseases. To this extend we utilized data sets that we previously generated for various tumors using the same technology and standard operating procedures. Specifically, we considered immunogenic antigens that were informative in the comparisons between glioma patients and controls, lung cancer patients and controls and prostate carcinoma patients and controls [27], [29], [32]. None of the clones that were highly informative for the separation between neuroblastoma and Wilms tumor including the clones for exostosin-2, ELAV-like protein 4, Microtubule-associated proteins 1A/1B light chain 3A precursor and amyloid beta A4 precursor protein-binding family B member 1 have been informative for any other separation. Few clones that were less but yet informative for the separation between neuroblastoma and Wilms tumor, were also informative for other classifications: Nonhistone chromosomal protein HMG-14 has also been informative for the classification between prostate carcinoma and controls and vimentin was also informative both for the classification between glioma and controls and prostate carcinoma and controls (data not shown).

In summary we identified an autoantibody response in neuroblastoma patients that showed a clearly increased number of immunogenic informative antigens compared to Wilms tumor patients. This is shown by the calculated AUC values for the classification accuracy. An AUC value smaller than 0.3 for an antigen means that this antigen reacted with significantly more sera from neuroblastoma patients than with sera from Wilms tumor patients whereas an AUC value higher than 0.7 for an antigen means that this antigen reacted with significantly more sera from Wilms tumor patients than with sera from neuroblastoma patients. As shown in Table 4 and 5, almost all antigens informative for the classification were more reactive with sera from neuroblastoma patients than with sera from Wilms tumor patients. The identified autoantibody response allows to the separate neuroblastoma from Wilms tumors with an accuracy of higher than 85%. Since none of the informative antigens has previously been reported as informative for other cancer patients, neuroblastoma appears to be a tumor with a rather specific and complex autoantibody response. It is tempting to speculate whether this response is related to the frequently observed spontaneous remission without any chemotherapy.

## Materials and Methods

### Study population

#### Neuroblastoma blood samples

Neuroblastoma blood samples were obtained with parents' informed consent from the Department of Pediatric Oncology and Hematology of the Medical School of Cologne. Serum was isolated and subsequently stored at −80°C. The study was approved by the ethics committee of the University of the Cologne on the 9^th^ of September 2004 for the study NB 2004. We used 60 sera from 30 neuroblastoma patients (two sera from each patient, one prior to and one after therapy). Sera from neuroblastoma patients have been collected between 1992 and 2003. Clinical data of the patients are shown in Figure S1.

#### Wilms tumor blood samples

Wilms tumor blood samples were obtained from the multicenter study SIOP 2001/GPOH. Serum was isolated and subsequently stored at −80°C. The study was approved by the local ethics committee of the “Ärztekammer des Saarlandes” on September 2010 for nephroblastoma study (No. 68/06). Patients or parents of minor patients gave their written consent. We used 53 sera from Wilms tumor patients prior to therapy and 35 sera of Wilms tumor patients after therapy. Sera of Wilms tumor patients have been collected between 2006 and 2009. Clinical data of the patients are shown in Figure S2 and S3.

### Protein macroarray screening

As previously described [29], we used a 1,827 clone array (imaGenes, Berlin, Germany) for analysis, derived from a high-density protein macroarray with 38,016 recombinant *E.coli* clones of the hex1 library [42]. We screened this array with sera of untreated and treated neuroblastoma patients and sera of untreated and treated Wilms tumor patients.

The 1,827 clones array was washed twice in TBSTT (TBS, 0.05% Tween20, 0.5% Triton X-100) and four times in TBS. Membranes were blocked with 3% non-fat dry milk in TBST (TBS, 0.05% Tween20) and incubated over night with diluted sera (1∶1000). The sera were stored for a second round of incubation. Membranes were washed with TBST and incubated with stripping solution at 70°C. Membranes were washed twice with TBST and four times with TBS and incubated in blocking solution for 2 h. Membranes were once more incubated with serum overnight. Membranes were washed with TBST and incubated with secondary antibody (rabbit anti-human IgG, IgA, IgM-Cy5 (H+L), Dianova, Hamburg, Germany) (1∶1000) for detection. Membranes were washed four times with TBST, twice with TBS, and dried overnight. Signal detection was carried out with a Typhoon 9410 scanner (GE Healthcare, Uppsala, Sweden).

### Image analysis

The spot intensity was evaluated by a fully automated image analysis procedure as previously described [43]. The image analysis of each filter resulted in an autoantibody profile consisting of 3,654 integer intensity values ranging from 0 (no signal) to 255 (maximal intensity), corresponding to the 1,827 clones measured in duplicates. The replicate values were averaged for all further analyses. Not-available values were assigned to clones where no appropriate spots could be detected. All 308 clones with more than ten not-available values were excluded from further analysis.

### Biostatistical analysis

To make the profiles from different arrays comparable to each other, we performed a standard quantile normalization to minimize array-to-array variations [43]. We used the remaining 1,520 clones for the classifications based on a linear kernel Support Vector Machine (SVM) as described previously [44]. To determine mean sensitivity, specificity and accuracy for the classification tasks, we performed 20 repetitions of a standard 10-fold cross validation and, additionally, 20 classification runs with randomly permuted class labels to test for overtraining. In addition, we computed the 95% confidence intervals (95% CI) for the classifications, the positive predicitive value (PPV) negative predicitive value (NPV) as well as the positive and negative likelihood ratios.

In order to determine the information content of single clones, we calculated the area under the receiver -operating -characteristic -curve (ROC) value (AUC value). The intensity values ranging from 0 to 255 were considered to study the discrimination potential of each seroreactive clone. For a given clone c and a threshold h, we considered Wilms tumor sera as true positive (TP) if clone c had an intensity value larger or equal h. If c had an intensity value smaller than h, the Wilms tumor sera were typed as false negative (FN). Neuroblastoma sera with intensity value above the threshold were considered as false positive (FP), sera with intensity values below the threshold were considered as true negative (TN). To calculate the ROC curve and the AUC value of the considered antigen, the sensitivity (TP/(TP+FN)) and specificity (TN/(TN+FP)) of varying thresholds were used. AUC values of either 0 or 1 indicate a perfect separation. So, we considered clones with AUC values below 0.3 and above 0.7 as informative for a given separation task. We considered normalized intensity values above 50 as positive seroreactivity to get binary information for each clone, i.e., whether the clone is absent or present in a sample.

## Supporting Information

Figure S1 **Clinical data of neuroblastoma patients (1/2 means 1 = prior to therapy, 2 = after therapy) with diagnosis and treatment.** Legend: 1p: 2 = no deletion; 4 = 1p deletion; 1,1 = imbalance; 1 = homozygous; n.d. = not done. MYCN: 1 = normal; >1 = amplification; n.d. = not done. Age: [days]. NSE: <20 = normal; >20 = pathological. Treatment: B = observation; HR = high risk.
(DOC)

Figure S2 **Clinical data of WT patients prior to treatment.** R = right, L = left, B = bilateral, CR = complete remission. Histology code is according to the “revised SIOP (Stockholm) working classification of renal tumors of childhood and adolescence”.
(DOC)

Figure S3 **Clinical data of WT patients after treatment.** R = right, L = left, B = bilateral, CR = complete remission. Histology code is according to the “revised SIOP (Stockholm) working classification of renal tumors of childhood and adolescence”.
(DOC)
