# Supplementary material for: Autoantibody Signature Differentiates Wilms Tumor Patients from Neuroblastoma Patients
Source: PLoS One. 2011 Dec 16;6(12):e28951. doi: 10.1371/journal.pone.0028951 (PMC3241697; doi:10.1371/journal.pone.0028951)
Supplement: Figure S1 — Clinical data of neuroblastoma patients (1/2 means 1 = prior to therapy, 2 = after therapy) with diagnosis and treatment. Legend: 1p: 2 = no deletion; 4 = 1p deletion; 1,1 = imbalance; 1 = homozygous; n.d. = not done. MYCN: 1 = normal; >1 = amplification; n.d. = not done. Age: [days]. NSE: <20 = normal; >20 = pathological. Treatment: B = observation; HR = high risk. (DOC) [file pone.0028951.s001.doc]

|  | sex | age | stage | treatment | MYCN | catecholamines | MIBG | 1p | NSE |
| --- | --- | --- | --- | --- | --- | --- | --- | --- | --- |
| Neuro2-1/2 | m | 190 | 1 | B | 1 | neg | path | 2 | 26,6 |
| Neuro3-1/2 | f | 587 | 1 | B | 1 | neg | path | n.d. | 14,1 |
| Neuro4-1/2 | m | 92 | 1 | B | 1 | VMA-U path | path | 2 | 18,5 |
| Neuro6-1/2 | f | 400 | 4 | HR | 1 | path | path | 2 | 11,5 |
| Neuro7-1/2 | m | 957 | 4 | diverse CT | 30 | HVA-S path | path | n.d. | 110 |
| Neuro8-1/2 | m | 2913 | 4 |  | 1 | path | path | n.d. | 25,9 |
| Neuro9-1/2 | f | 3151 | 4 | HR | n.d. | n.d. | path | n.d. | 876 |
| Neuro10-1/2 | f | 1481 | 4 | HR | 1 | path | path | n.d. | 74,5 |
| Neuro11-1/2 | m | 132 | 1 | B | 1 | path | path | n.d. | 30,5 |
| Neuro13-1/2 | f | 439 | 1 | B | 1 | neg | n.d. | 2 | 8 |
| Neuro14-1/2 | m | 75 | 1 | B | 1 | neg | neg | n.d. | 7,4 |
| Neuro15-1/2 | f | 494 | 1 | B | 1 | HVA-U path | neg | 2 | 22,7 |
| Neuro17-1/2 | m | 611 | 1 | B, 3d Wilms CT | 1 | path | path | 2 | 65 |
| Neuro19-1/2 | f | 3984 | 1 | B | 1 | neg | neg | n.d. | 11,9 |
| Neuro20-1/2 | f | 300 | 1 | B | 1 | neg | neg | 2 | 37,3 |
| Neuro22-1/2 | m | 456 | 1 | B | 1 | path | path | 2 | k.a. |
| Neuro23-1/2 | m | 1598 | 1 | B | 1 | path | path | 1 | 21,4 |
| Neuro24-1/2 | m | 216 | 1 | B | 1 | path | path | 4 | k.a. |
| Neuro26-1/2 | m | 476 | 4 | HR | 1 | path | path | 2 | 27,4 |
| Neuro27-1/2 | f | 388 | 4 | HR | 1 | path | path | 2 | 38,5 |
| Neuro30-1/2 | m | 2059 | 4 | HR | 20 | HVA-U+S path | path | 4 | 32,6 |
| Neuro31-1/2 | m | 1712 | 4 | HR | n.d. | path | n.d. | n.d. | 135 |
| Neuro32-1/2 | f | 1017 | 4 | HR | 1 | path | path | 4 | 67,9 |
| Neuro33-1/2 | m | 1587 | 4 | HR | 1 | neg | path | 2 | 200 |
| Neuro34-1/2 | m | 106 | 4 | HR | 1 | path | path | 2 | 184,5 |
| Neuro35-1/2 | f | 1221 | 4 | HR | 1 | path | path | 1,1 | k.a. |
| Neuro36-1/2 | f | 2767 | 4 | HR | 1 | path | path | 2 | k.a. |
| Neuro37-1/2 | m | 724 | 4 | HR | 1 | path | path | 4 | 214 |
| Neuro38-1/2 | m | 366 | 4 | HR | 1 | path | path | 1,1 | 60,4 |
| Neuro39-1/2 | m | 840 | 4 | HR | 1 | path | path | n.d. | 149,9 |
