# Supplementary material for: Autoantibody Signature Differentiates Wilms Tumor Patients from Neuroblastoma Patients
Source: PLoS One. 2011 Dec 16;6(12):e28951. doi: 10.1371/journal.pone.0028951 (PMC3241697; doi:10.1371/journal.pone.0028951)
Supplement: Figure S2 — Clinical data of WT patients prior to treatment. R = right, L = left, B = bilateral, CR = complete remission. Histology code is according to the “revised SIOP (Stockholm) working classification of renal tumors of childhood and adolescence”. (DOC) [file pone.0028951.s002.doc]

|  | **GENDER** | **Age [days]** | **Histology** | **Local Stage** | **Localiasation of tumour** | **Regression L** | **Regression R** | **Metastasis at date of diagnosis?** | **Location of Metastasis** | **Blastemal subpopulation** | **Ephitelial subpopulation** | **Stromal subpopulation** | **Status** |
| --- | --- | --- | --- | --- | --- | --- | --- | --- | --- | --- | --- | --- | --- |
| Hom57-1 | f | 601 | 5 | I | L | <65% |  | None |  | 1% | 29% | 70% | CR |
| Hom1-1 | m | 1441 | 6 | I | R |  | 50% | None |  |  | 50% | 50% | CR |
| Hom111-1 | m | 1223 | 7(L+R) | II (L),I (R) | B | 100% | 95% | yes | lung |  |  |  | CR |
| Hom112-1 | f | 8 | 9 | I | R |  | <65% | None |  | 95% | 5% | 0 | CR |
| Hom12-1 | f | 1646 | 3 (L + R) | III (R), L? | B | 100% |  | None |  |  |  |  | CR |
| Hom121-1 | f | 2024 | 4 | II | L |  | 20% | None |  | 0 | 100% | 0 | Progression |
| Hom126-1 | f | 2788 | 7 | I | R |  | 99% | yes | lung | 0 | 0 | 100% | CR |
| Hom128-1 | m | 172 | 7 | I | L | 95% |  | None |  | 0 | 30% | 70% | CR |
| Hom132-1 | m | 700 | 6 | III | R |  | <65% | None |  | 50% | 20% | 30% | CR |
| Hom133-1 | f | 286 | 5 | I | R |  | <65% | None |  | 10% | 0 | 90% | CR |
| Hom134-1 | f | 3113 | 7 | I | L | 98% |  | yes | lung | 0 | 5% | 95% | CR |
| Hom144-1 | m | 238 | 5 | I | R |  | 25% | None |  | 0 | 0 | 100% | CR |
| Hom16-1 | f | 1000 | 7 | II | L | 70% |  | None |  | 0 | 50% | 50% | CR |
| Hom17-1 | f | 1523 | 6 | II | R |  | 5% | None |  | 0 | 60% | 40% | CR |
| Hom21-1 | f | 809 | 6 | III | R |  | 20% | None |  | 1% | 50% | 49% | CR |
| Hom22-1 | f | 2344 | 9 | III | R | 60% |  | None |  |  |  |  | CR |
| Hom23-1 | m | 900 | 7 | II | R |  | 65-99% | None |  |  |  |  | CR |
| Hom25-1 | f | 1233 | 7 | III | R |  | 70% | None |  | 10% | 50% | 40% | CR |
| Hom27-1 | f | 327 | 6 | I | R |  | 10% | None |  | 15% | 60% | 35% | CR |
| Hom31-1 | m | 278 | 6 | I | L | 25% |  | None |  | 0 | 70% | 30% | CR |
| Hom36-1 | f | 1434 | 7 | II | R |  | 80% | None |  | 100% | 0 | 0 | CR |
| Hom4-1 | m | 2539 | 6 | I | L | 60% |  | None |  | 40% | 30% | 30% | CR |
| Hom41-1 | m | 1014 | 6 | I | R |  | 15% | None |  | 30% | 30% | 40% | CR |
| Hom42-1 | m | 778 | 7 | II | L | 30% |  | None |  | 40% | 30% | 30% | CR |
| Hom43-1 | f | 696 | 7 | I | L | 99% |  | None |  |  |  |  | CR |
| Hom46-1 | f | 1404 | 6 (R) 15 (L) | I (L), R? | B | 0% |  | None |  | 20% | 5% | 75% | CR |
| Hom50-1 | m | 2126 | 6 | I | R |  | 30% | yes | lung |  |  |  | CR |
| Hom51-1 | m | 1408 | 6 | II | R |  | 40% | None |  | 10% | 30% | 60% | CR |
| Hom52-1 | m | 1425 | 5 | I | R |  | 30% | None |  | 10% | 20% | 70% | CR |
| Hom74-1 | m | -207 | 7 | I | R |  | 95% | None |  | 0 | 70% | 30% | CR |
| Hom75-1 | f | 2943 | 7 | II | L | 80% |  | None |  | 0 | 60% | 40% | CR |
| Hom77-1 | f | 2992 | 4 | I | L | 30% |  | None |  | 5% | 85% | 10% | CR |
| Hom78-1 | m | 1402 | 10 (L + R) | III (R) , II (L) | B | 60% | 90% | None |  | 100% |  |  | DEAD |
| Hom80-1 | f | 1763 | 6 | I | R |  | 40% | None |  | 98% | 2% | 0 | CR |
| Hom81-1 | m | 436 | 6 | II | L | 35% |  |  |  |  |  |  | CR |
| Hom88-1 | f | 957 | 6 | II | R |  | 60% | None |  | 0 | 5% | 95% | CR |
| Hom90-1 | f | 79 | 6 | I | L |  | 5% |  |  | 50% | 45% | 5% | CR |
| Hom93-1 | f | 937 | 5 | II | L | 5% |  | None |  | 5% | 5% | 90% | CR |
| Hom94-1 | f | 1818 | 7 | II | R |  | 90% | None |  | 0 | 2% | 98% | CR |
| Hom95-1 | f | 1826 | 6 | III | L | 35% |  | None |  | 0 | 40% | 60% | CR |
| Hom97-1 | m | 885 | 10 | I | R |  | 60% | None |  | 100% | 0 | 0 | CR |
| Hom98-1 | f | 635 | 4 | I | R |  | 0 | None |  | 5% | 85% | 10% | CR |
| hom159-1 | f | 867 | 6 | I | L | 10% |  | None |  | 20% | 30% | 50% | CR |
| hom160-1 | f | 1185 | 6 | II | R |  | 5% | None |  | 70% | 20% | 10% | CR |
| hom179-1 | m | 294 | 4 | I | L | 5% |  | None |  | 0 | 100% | 0 | CR |
| hom181-1 | f | 778 | 7 | I | L | 80% |  | None |  | 80% | 20% | 0 | CR |
| hom185-1 | f | 1302 | 7 | I | L | 99% |  | None |  | 100% |  |  | CR |
| hom194-1 | f | 824 | 7 | III | R |  | 80% | None |  | 0 | 100% | 0 | CR |
| hom195-1 | m | 3797 | 7 | III | L | 92% |  | yes | lung, liver, LN | 2% | 8% | 90% | DEAD |
| hom198-1 | m | 436 | 9 | III | R |  | 40% | None |  |  |  |  | CR |
| hom5-1 | f | 353 | 7 | I | R |  | 80% | None |  | 80% | 10% | 10% | CR |
| hom64-1 | f | 49 | 4 | I | R |  | 0 | None |  | 5 | 85 | 5 | CR |
| hom8-1 | m | 2710 | 3 | I | L | 100% |  | yes | liver |  |  |  | CR |
