# Supplementary material for: Autoantibody Signature Differentiates Wilms Tumor Patients from Neuroblastoma Patients
Source: PLoS One. 2011 Dec 16;6(12):e28951. doi: 10.1371/journal.pone.0028951 (PMC3241697; doi:10.1371/journal.pone.0028951)
Supplement: Figure S3 — Clinical data of WT patients after treatment. R = right, L = left, B = bilateral, CR = complete remission. Histology code is according to the “revised SIOP (Stockholm) working classification of renal tumors of childhood and adolescence”. (DOC) [file pone.0028951.s003.doc]

| **W-Nummer** | **GENDER** | **Age [days]** | **Histology** | **Local Stage** | **Lokalisation Tumor** | **Regression L** | **Regression R** | **Metastasis at date of diagnosis?** | **Location of Metastasis** | **Blastemal subpopulation** | **Ephitelial subpopulation** | **Stromal subpopulation** | **Status** |
| --- | --- | --- | --- | --- | --- | --- | --- | --- | --- | --- | --- | --- | --- |
| w10 | f | 384 | 7 | I | R |  | 80% | None |  | 80% | 10% | 10% | CR |
| w11 | m | 1907 | 5 | I | R |  | 5% | None |  | 0 | 30% | 70% | CR |
| w12 | f | 726 | 10 | I | R |  | 50% | None |  | 90% | 5% | 5% | CR |
| w13 | f | 861 | 6 | I | L | 50% |  | None |  | 25% | 25% | 50% | CR |
| w14 | m | 1296 | 10 | I | L | 70% |  | None |  | 95% | 3% | 2% | CR |
| w15 | f | 2774 | 3 | I | L | 100% |  | yes | liver | 0 | 0 | 0 | CR |
| w16 | f | 1039 | 7 | II | L | 70% |  | None |  | 0 | 50% | 50% | CR |
| w17 | f | 455 | 5 + ACC - CA | I | B |  |  | None |  |  |  |  | CR |
| w18 | f | 838 | 6 | III | R |  | 20% | None |  | 1% | 50% | 49% | CR |
| w19 | f | 2098 | 10 | III | R |  | 0% | yes | lung, liver | 100% | 0 | 0 | DEAD |
| w1 | f | 558 | 6 | I | L | <65% |  | None |  |  |  |  | CR |
| w20 | m | 912 | 7 | II | R | 65-99% |  | None |  |  |  |  | CR |
| w21 | m | 1511 | 7 | I | L | 80% |  | None |  | 100% |  |  | CR |
| w23 | f | 1786 | 7 | I | L | 80% |  | None |  |  |  | 100% | CR |
| w24 | m | 775 | 3 | III | L | 100% |  | yes | lung |  |  |  | CR |
| w28 | m | 2746 | 6 | II | R |  | 10% | None |  | 40% | 30% | 30% | Progression |
| w2 | f | 535 | 4 | I | L | 20% |  | None |  |  | 50% | 50% | CR |
| w30 | f | 1004 | 5 | I | L | 70% |  | None |  | 5% | 15% | 80% | CR |
| w31 | f | 1471 | 7 | II | R |  | 80% | None |  | 100% |  |  | CR |
| w32 | m | 3594 | 4 | I | L | 50% |  | None |  | 90% | 10% |  | CR |
| w33 | f | 292 | 4 | II | L | 15% |  | None |  | 0 | 80% | 20% | CR |
| w34 | m | 1448 | 7(R) and 6 (L) | I (R), II (L) | B | 15% | 98% | yes | LN | 100% (R), 25% (L) | 30% (L) | 25% (L) | CR |
| w35 | m | 280 | 4 (L + R) | I (L+R) | B | 15% | 1% | None |  |  | 100% (L and R) |  | Partial Remission/Residual Disease |
| w36 | m | 853 | 5 | II | R |  | 50% | None |  |  | 2% | 98% | CR |
| w37 | m | 4650 | 7 | I | R |  | 80% | None |  | 95% | 5% | 0 | CR |
| w38 | f | 1972 | 6 | I | R |  | 10% | None |  | 64% | 36% | 0 | CR |
| w39 | m | 1454 | 6 | II | R |  | 40% | None |  | 10% | 30% | 60% | CR |
| w41 | m | 1457 | 5 | I | R |  | 30% | None |  | 10% | 20% | 70% | CR |
| w42 | f | 1474 | 15 (R) and 6 (L) | I (L + R) | B | 0 |  | None |  | 20% (L) | 5% (L) | 75% (L) | CR |
| w4 | m | 3899 | 10 | III | L | 50% |  | yes | lung |  |  |  | DEAD |
| w5 | m | 7134 | 6 | III | R |  |  | None |  |  |  |  | DEAD |
| w6 | f | 1578 | 7 | I | L | 70% |  | yes | lung | 40% | 10% | 50% | CR |
| w7 | f | 3120 | 7 | III | R |  | 98% | None |  | 0 | 100% | 0 | CR |
| w8 | m | 2572 | 6 | I | R |  | 70% | None |  | 90% | 0 | 10% | CR |
| w9 | f | 4006 | 3 | II | R |  | 100% | None |  | 0 | 0 | 0 | CR |
